# Chemical Composition of Essential Oils of Aromatic and Medicinal Herbs Cultivated in Greece—Benefits and Drawbacks

**DOI:** 10.3390/foods10102354

**Published:** 2021-10-03

**Authors:** Ioannis N. Pasias, Dimitris D. Ntakoulas, Kalomoira Raptopoulou, Chrysavgi Gardeli, Charalampos Proestos

**Affiliations:** 1 General Chemical Lab of Research and Analysis, 35100 Lamia, Greece; iopas@chem.uoa.gr (I.N.P.); dimitrisntakoulas@gmail.com (D.D.N.); kalirapt@chem.uoa.gr (K.R.); 2 Food Chemistry Laboratory, Department of Chemistry, National and Kapodistrian University of Athens, 15772 Athens, Greece; 3 Food Chemistry and Analysis Laboratory, Department of Food Science and Human Nutrition, Agricultural University of Athens, 11855 Athens, Greece; agardeli@aua.gr

**Keywords:** antioxidant, nutritional aspects, GC-FID, GC-MS, allergic reactions, antimicrobial activity

## Abstract

The current study investigated and determined the major compounds of six essential oils derived from different plant species cultivated in Greece (*Lavandula angustifolia, Origanum vulgare, Pistacia lentiscus* var. *chia, Citrus reticulata, Citrus limon* and *Crithmum maritimum*). The results proved that all these essential oils have a high potential for use as food preservatives, since among the compounds determined were sabinene, b-myrcene, a-pinene, carvacrol and limonene, all of which were responsible for the strong antimicrobial activity against *Staphylococcus aureus*. However, the amounts of some compounds, such as linalool and citral, were at high levels, and this can be a danger for some sensitive population groups due to allergic reactions. The essential oil compounds which were identified using GC-MS and quantified through GC-FID represented more than 95% of the total essential oils of the investigated plant species. Finally, all essential oils provided high phenolic content.

## 1. Introduction

In the last few decades, chemical and synthetic preservatives have been widely used to conserve the quality and extend the shelf life of food products [1]. However, chemical preservatives can have negative effects on food safety, and may damage consumers′ health due to their carcinogenic and teratogenic effects or residual toxicity [2,3]. Therefore, natural compounds must be adopted to ensure the freshness and quality of the foods [4]. Essential oils are among those natural compounds that are used nowadays to prolong the shelf life of food products, because they show strong antioxidant and antimicrobial activity [5].

Essential oils are volatile odoriferous oils and secondary metabolites obtained from the volatile part of aromatic plants, and are used by the plants as a defense mechanism against herbivore attacks [6]. The essential oils can be obtained from aromatic plants through different methods including expression, steam distillation extraction, reflux extraction and Soxhlet extraction [7]. They are used extensively in cosmetics and medicine due to their sweet-smelling odors and pharmacological properties [8]. Furthermore, the essential oils of many plant species show antioxidant [9], antiproliferative [10] and antimicrobial activity. They can also be used as substitutes to antibiotics [11], and have proven to be effective in a wide range of applications by lessening the growth of pathogen microorganisms [12], or by providing promising activity against antibiotic-resistant bacteria and chemotherapeutic-resistant tumors [13]. Among the most widely used essential oils for the food and cosmetics industry are *Lavandula angustifolia* Mill., *Origanum vulgare* L., *Pistacia lentiscus* L. var. *chia, Citrus reticulata* L., *Citrus limon* L. and *Crithmum maritimum* L.

*L. angustifolia* (Lamiaceae) is a medicinal and aromatic plant. This herb is used around the world in both conventional and folk medicines due to its anti-inflammatory and analgesic properties. This herb is among the most cultivated species in the Mediterranean region, and is most often used in the production of cosmetics, perfumes and essential oils. In the last few years, it has also been used in the food industry as a natural flavoring agent [14,15,16]. *O. vulgare* is a herb that also belongs to the Lamiaceae family, and is widely used as a culinary herb in gastronomy [17]. It is also used in traditional medicine to cure dyspepsia, rheumatoid arthritis, scrofulosis and respiratory and urinary tract disorders. There are studies that report the use of oregano essential oil as a preservative in the food industry [18,19]. The antioxidant effect of oregano essential oil is associated with some of its major ingredients, such as carvacrol [20]. *P. lentiscus* var. *chia* is cultivated in Chios, a Greek island of the eastern Aegean Sea, and produces mastiha, an aromatic resin in a teardrop shape. *P. lentiscus* var. chia essential oil is produced from the flowers, leaves and branches of mastic trees. Extracts obtained from the mastic tree are used in traditional medicine to treat diseases such as eczema, throat infections, dyspepsia and gastralgia, because they contain substances with antiseptic and anti-inflammatory properties [21]. The major compounds of *P. lentiscus* var. chia essential oil are a-pinene, β-myrcene, β-pinene and limonene [22].

The *Citrus* genus belongs to the *Rutaceae* family, and has characteristic odors and pleasurable flavors. The mandarin (*C. reticulata*) and lemon (*C. limon*) both belong to this family, and their essential oils exhibit high antioxidant activity as well as numerous medicinal and aromatherapeutic properties, such as analgesic, anti-anxiety, antispasmodic and chemoprotective properties [23]. In addition, essential oils of lemon and mandarin are used as food additives in order to inhibit the growth of some bacteria and prevent food spoilage [24]. Finally, *C. maritimum*, also known as sea fennel, is an aromatic plant cultivated on rocky sea cliffs. This plant is very important for nutrition and contains many medicinal applications due to its tonic, diuretic and anti-inflammatory properties [25,26]. The essential oil of *C. maritimum* shows high antioxidant and antibacterial activity, and has been used as a preservative in food products [27].

Hence, the aim of this work was to study, report and associate the composition of the essential oils of lavender *(L. angustifolia),* oregano *(O. vulgare),* mastic tree *(P. lentiscus* var. *chia*), lemon *(C. limon),* mandarin *(C. reticulata),* and sea fennel *(C. maritimum*) that are cultivated in different prefectures of Greece, with the possibility of use for food preservation, and to indicate the possible drawbacks of their use on special groups of the population. For this reason, a GC-FID method was applied for the determination of the major compounds, and GC-MS was also used for the comparison and characterization of compounds. More than 95% of the peaks were characterized and quantified. Furthermore, the antimicrobial and antioxidant activities were investigated by determining the minimum lethal concentration (MLC) and total phenolic content (TPC) of the six essential oils investigated.

## 2. Materials and Methods

In the current work, 6 essential oils were derived from *L. angustifolia, O. vulgare, P. lentiscus* var. *chia, C. reticulata, C. limon* and *C. maritimum*. All samples were provided from local farmers in different regions of Greece and transferred as ready-to-use samples in the laboratory. Essential oils were isolated using only typical distillation procedures. More specifically, *L. angustifolia* and *O. vulgare* essential oil samples were purchased from local producers in the prefecture of Fthiotida, Greece, *P. lentiscus* var. *chia* essential oil samples were purchased from Chios island, Greece, *C. reticulata* and *C. limon* essential oil samples were collected from the prefecture of Argolida Greece and *C. maritimum* samples were collected from the prefecture of Magnesia, Greece. All samples had lot numbers on them to provide traceability, and five different samples from each specimen were used.

All samples were analyzed using a Shimadzu gas chromatography (GC) system, equipped with a FID detector and DB-5 capillary column (30 m × 0.25 mm; film thickness, 0.25 μm). The injector and detector temperatures were set at 220 and 260 °C, respectively. The initial GC oven temperature was set at 60 °C, where it was held for 12 min, then programmed from 60 to 165 °C at a rate of 5 °C/min, and then to 250 °C at a rate of 10 °C/min where it was held isothermally for 12 min. Helium was the carrier gas at a flow rate of 3 mL/min. A total of 1.0 μL of diluted samples (1/100 in hexane) was injected manually and in the spitless mode. Quantitative data were obtained using the peak area mode. For quantification and peak identification, different reference solutions were purchased from Sigma-Aldrich (Steinheim, Germany).

GC-MS analysis was also used for the identification of the major compounds (Table 1) of all samples analyzed with a Finnigan Trace GC Ultra 2000 (Thermo Electron Corporation, Waltham, MA, USA) gas chromatography system, equipped with a mass spectrometry detector and a Trace TR-5MS (Thermo Scientific, Waltham, MA, USA) column. The injector and detector temperatures were set at 220 and 250 °C, respectively. The initial GC oven temperature was set at 50 °C where it was held for 3 min, then programmed from 50 to 210 °C at a rate of 3 °C/min and then to 240 °C at a rate of 15 °C/min where it was held isothermally for 2 min. The ionization mode used was Electron Impact (EI), and the detector voltage was set at 70 eV. Helium was the carrier gas at a flow rate of 1 mL/min. A total of 1.0 μL of diluted samples (1/1000 in hexane) was injected and in the spitless mode.

The Spectrophotometric Folin–Ciocalteu method was used for the determination of total phenolic content (TPC) in all different essential oils using a Hach Lange DR6000 UV-visible spectrometer. More specifically, 100 µL of the diluted solution in ethanol essential oil (1:10) was mixed with 100 µL of the Folin–Ciocalteu reagent, and 3.5 mL of purified water and 300 µL of a 20% (*w/w*) solution of sodium carbonate were added. The mixture was mixed by vortex and incubation was performed for 120 min at room temperature in darkness. The absorbance was read at 760 mm. Purified water was used as the blank sample. The test was carried out for each essential oil in triplicate. The results were expressed as mg gallic acid equivalents (GAE) per g of essential oil, and are presented in Table 2.

The minimum inhibitory concentration or minimum lethal concentration (MLC) was used for the determination of the antimicrobial activity of all essential oils versus *S. aureus* (ATCC 13563), as described in Elgayyar et al. work (2001) [28]. The essential oil concentration ranged between 100 and 50,000 ppm. MLC cultures were incubated at 37 °C for 48 h. Each sample was prepared in triplicate and the results are presented in Table 2. A blank sample was also prepared in triplicate to set the minimum percentage of inhibition.

## 3. Results

The percentage composition and the compounds identified from the essential oil analysis are presented in Table 1.

**Figure 1:**
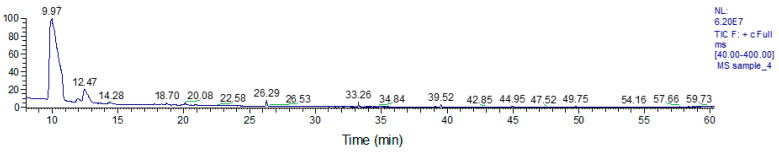
GC-MS chromatogram of the *P. lentiscus* var. *chia* essential oil analysis.

A GC-MS analysis was also used for the identification of the major compounds for all essential oils tested. The majority of components were identified (more than 95%) for all samples tested. Figure 1 shows the chromatogram of the *P. lentiscus* var. *chia* essential oil by the GC-MS analysis. The peak at 9.97 min corresponds to alpha-pinene, whereas at 12.47 beta-myrcene appears.

In Table 2, the results of the antimicrobial and antioxidant activity are presented for all essential oils investigated in the current study. The results are presented as the mean value minus/plus the standard deviation of three independent determinations.

## 4. Discussion

According to the results obtained, in the *L. angustifolia* essential oil, linalyl acetate (30.05%) and linalool (27.85%) were the most abundant components (Table 1). According to the ISO 3515 standard, the oil should contain linalyl acetate (25–47%), linalool (max. 45%), terpinen-4-ol (max. 8%), camphor (max. 1.5%), limonene (max. 1%) and 1,8-cineole (max. 3%) [29]. The results are in accordance with the ISO standard [28]. Even though lavender essential oil is known for its benefits of expanding the shelf-life of foods and for aromatherapy reasons including the relief of the symptoms of stress, depression and anxiety [30], it contains a high percentage of linalool and limonene, which are both part of the list of 26 allergenic fragrances. These compounds cause a serious allergic contact dermatitis, as provided by De Groot and Schmidts’ (2016) work, in which they summarized 19 publications on allergic contact dermatitis caused by lavender oil [31]. In some of these case reports, the patients allergic to lavender oil also showed positive patch test results to linalool. However, this high amount of linalool was proved to provide strong antimicrobial activity against *Staphylococcus aureus* and *Escherichia coli*, expanding the shelf life of many vegetables and dairy products that are rich in these microorganisms [4]. More specifically, in the current work, it was proved that the minimum lethal concentration for *L. angustifolia* essential oil against *S. aureus* was 1500 ppm. Furthermore, it was proved that *L. angustifolia* essential oil is a rich source of phenols, providing a TPC of 25.3 mgGAE/g (Table 2).

In the case of *O. vulgare*, essential oil analysis revealed two main compounds, carvacrol (74.2%) and p-cymene (8.2%). Carvacrol appears to have high antimicrobial potential due to the presence of a free hydroxyl group, and other physical properties such as hydrophobicity. It is proved to be very effective against foodborne pathogens, including *E. coli*, *Salmonella* and *B. cereus*, inhibiting their growth and the production of toxins, as well as providing antifungal protection [32]. In the current work, it was also proved that *O. vulgare* essential oil provides the strongest antimicrobial activity against *S. aureus*, among all the different essential oils analyzed. The strong antimicrobial activity is also related to the high content of p-cymene, a compound that has been shown to have many biological actions such as anti-inflammatory, anticancer, antimicrobial and antioxidant effects, but is not yet validated for its safe use against all these diseases [33]. Concerning the main allergen content, it was proved that the current samples did not contain linalool and citral, but they possessed a small content of limonene (about 0.2% *w/w*). Therefore, the high content of carvacrol (74.2%) and p-cymene found in the current work proves that oregano essential oil can be used, and is a valuable method for natural food preservation and for preventing food spoilage and microbial growth, especially since it has a food-friendly odor. Finally, *O. vulgare* essential oil proved to be a good source of phenols, providing the highest TPC among all the other essential oils tested.

The citrus species *C. reticulata* and *C. limon* provided similar profiles according to their similar geographical origin and family. More specifically, in both essential oils limonene was the main compound, with a percentage higher than 71% (*w/w*), followed by pinenes and gamma-terpinene. The high limonene content provides both essential oils an attractive flavor and fragrance, and therefore it has many applications in the cosmetic, food and beverage industries. Current studies, as presented in the review of Ibáñez et al. (2020), have proved that limonene also provides strong protection against food-spoilage microorganisms, as well as antioxidant potential to avoid post-harvest decay in the storage/packaging processes, thus extending the shelf-life of food products [34]. In the current work, it was also proved that citrus species, particularly *C. limon,* provide a strong antimicrobial activity against *S. aureus*. However, limonene is a highly allergic compound causing dermatitis, especially when exposed to air-producing sub-products [35]. Several studies examined the potential allergic risks from limonene, since both essential oils are used in many cosmetics and hand cleaners to provide aroma. The percentage of persons that were allergic to these compounds ranged from 4 to 10% [36,37,38,39]. Furthermore, *C. limon* contains a high amount of citral (2.5%) that can also cause severe dermatitis [39]. Finally, both species provided a similar TPC, as presented in Table 2.

The major ingredient of *P. lentiscus* var. *chia* was alpha-pinene (73.2%), followed by beta-myrcene (21.2%). A total of 13 compounds were detected by the GC-FID and identified by a GC-MS analysis. In general, as provided in similar works, the mastic essential oil can provide 34 compounds [22], where the main components are a-pinene and b-myrcene in a content ratio of two to four, depending on the method of determination [40]. In Gardeli et al. (2008), it is stated that alpha-pinene is also the major essential oil constituent of the variety *P. lentiscus* [41]. These major components play an important role in the ability of the mastic gum essential oil to prevent the growth of *Staphylococcus aureus*, *Lactobacillus plantarum*, *Pseudomonas fragi* and *Salmonella enteritidis*. In Tassou and Nychas (1995), it was proved that the rate of inhibition was greater in Gram-positive bacteria than that observed in Gram-negative bacteria [3]. In the current work, *P. lentiscus* var. *chia* provided an MLC value of 2350 ppm, and a total phenolic content of 12.6 mgGAE/g. Furthermore, it is important to note that the current essential oil was low in allergens, and can be safely used in food preservation. The only allergen compound that was detected was linalool, with a small content of 0.15% (*w/w*).

Concerning the *C. maritimum* essential oil, sabinene (49.45%) and gamma-terpinene (31.37%) were revealed as the major compounds, followed by alpha-pinene. The content of these compounds was high, especially the sabinene content [42]. These compounds are mainly used to improve the stability of roasted vegetables or seeds, preventing lipid oxidation and the development of rancid odors and tastes [43]. Furthermore, *C. maritimum* essential oil contains, in lower amounts, other bioactive compounds used in food preservation, such as beta-myrcene (1.1%), and p-cymene (0.55%). It also contains limonene (2.73%), which has an antifungal activity; however, this is contained in the list of 26 allergenic fragrances. These percentages were not able to provide a strong MLC value against *S. aureus,* and the total phenolic content was the lowest among the essential oils tested.

## 5. Conclusions

The current work proved that the essential oils of *L. angustifolia, O. vulgare, P. lentiscus* var. *chia, C. reticulata, C. limon* and *C. maritimum* contain a large amount of major and minor components that can be used in the food industry as preservatives, since they provide a significant defense against the growth of *Staphylococcus aureus*. More than 95% of the components were determined and quantified using the GC-FID method, and the peaks were also characterized using a GC-MS technique. The main components that have an important nutritional aspect are sabinene, gamma-terpinene, beta-myrcene, carvacrol and p-cymene. Furthermore, *O. vulgare* provided the highest antimicrobial and antioxidant activity among all essential oils investigated. All essential oils demonstrated significant antioxidant activity, with a TPC content that ranged from 7.5 to 42.6 mgGAE/kg. On the other hand, major components such as limonene, linalool and citral have some drawbacks in their use, since they can cause serious allergic reactions. As such, there needs to be more research regarding their use as food preservatives.

## Figures and Tables

**Table 1:** Chemical composition of six essential oils analyzed by GC-FID.

Compound			*Lavandula angustifolia* Mill.	*Origanum vulgare* L.	*Citrus reticulata* L.	*Citrus limon* L.	*Pistacia lentiscus* L. var. *Chia*	*Crithmum maritimum* L.
	Retention Time (Min)	RI	% (*w/w*)					
alpha-thujene	7.91	923	0.14 ± 0.03	0.12 ± 0.01	n.d.	n.d.	n.d.	0.28 ± 0.05
pinenes	8.13	939	0.32 ± 0.01	2.01 ± 0.15	3.76 ± 0.43	12.7 ± 0.21	73.2 ± 2.19	9.57 ± 002
1-octen-3-ol	8.17	940	0.21 ± 0.02	0.32 ± 0.05	n.d.	n.d.	n.d.	n.d.
camphene	8.29	953	0.15 ± 0.01	0.61 ± 0.03	n.d.	n.d.	1.16 ± 0.21	n.d.
sabinene	8.47	974	0.03 ± 0.005	n.d.	n.d.	1.02 ± 0.04	1.18 ± 0.11	49.45 ± 1.78
1,8-cineol	10.03	983	0.84 ± 0.07	n.d.	n.d.	n.d.	n.d.	n.d.
3-octanone	10.70	984	0.67 ± 0.03	n.d.	n.d.	n.d.	n.d.	n.d.
beta-myrcene	10.66	991	1.13 ± 0.07	1.29 ± 0.21	2.56 ± 0.34	1.47 ± 0.12	21.2 ± 1.22	1.06 ± 0.06
alpha-phellandrene	11.00	1000	0.22 ± 0.04	n.d.	n.d.	n.d.	0.31 ± 0.02	n.d.
alpha-terpinene	11.91	1017	0.09 ± 0.01	5.18 ± 0.43	n.d.	n.d.	0.75 ± 0.03	0.78 ± 0.03
alpha-terpinolene	12.05	1019	0.14 ± 0.01	0.12 ± 0.01	0.23 ± 0.02	0.31 ± 0.04	n.d.	n.d.
p-cymene	12.20	1027	0.12 ± 0.03	8.2 ± 0.45	0.21 ± 0.01	0.15 ± 0.01	n.d.	0.55 ± 0.06
beta-phellandrene	12.70	1029	0.15 ± 0.03	n.d.	n.d.	n.d.	0.12 ± 0.01	n.d.
limonene	12.95	1031	0.37 ± 0.01	0.21 ± 0.04	74.0 ± 2.23	71.7 ± 1.25	0.62 ± 0.01	2.73 ± 0.04
gamma-terpinene	14.08	1059	0.26 ± 0.03	5.78 ± 0.31	14.7 ± 1.04	8.52 ± 1.01	0.12 ± 0.01	31.37 ± 3.21
ocimene	14.20	1060	10.77 ± 0.49	n.d.	n.d.	n.d.	n.d.	n.d.
geranyl acetate	14.50	1094	0.94 ± 0.04	n.d.	n.d.	n.d.	0.28 ± 0.01	n.d.
linalool	14.71	1098	27.85 ± 1.21	n.d.	n.d.	n.d.	0.15 ± 0.01	n.d.
1-octen-3 yl acetate	17.22	1110	1.20 ± 0.09	n.d.	n.d.	n.d.	n.d.	n.d.
lavandulol	19.12	1157	0.88 ± 0.05	n.d.	n.d.	n.d.	n.d.	n.d.
terpinen-4-ol	20.55	1179	6.4 ± 0.21	1.8 ± 0.08	n.d.	n.d.	n.d.	1.50 ± 0.37
camphor	20.79	1181	0.19 ± 0.01	n.d.	n.d.	n.d.	0.11 ± 0.01	n.d.
alpha-Terpineol	21.00	1184	1.62 ± 0.51	n.d.	n.d.	n.d.	n.d.	n.d.
Citral	21.13	1240	n.d.	n.d.	n.d.	2.53 ± 0.43	n.d.	n.d.
linalyl acetate	21.35	1257	30.05 ± 0.12	n.d.	n.d.	0.50 ± 0.03	n.d.	n.d.
lavandulyl acetate	21.70	1290	4.3 ± 0.09	n.d.	n.d.	n.d.	n.d.	n.d.
Carvacrol	21,93	1298	n.d.	74.2 ± 1.7	n.d.	n.d.	n.d.	n.d.
beta caryophyllene	22.47	1428	3.81 ± 0.17	n.d.	n.d.	n.d.	0.19 ± 0.01	n.d.
trans-beta-farnesene	22.72	1454	3.26 ± 0.32	n.d.	n.d.	n.d.	n.d.	n.d.
Total identified compounds			96.11	99.84	95.46	98.9	99.39	97.29

**Table 2:** Minimum lethal concentration (MLC) (*n* = 3) and total phenolic content (TPC) (*n* = 3) of the six essential oils analyzed.

Essential Oil	MLC (ppm)	TPC (mgGAE/g)
*Lavandula angustifolia* Mill.	1500 ± 120	25.3 ± 1.4
*Origanum vulgare* L.	510 ± 63	42.6 ± 3.9
*Citrus reticulata* L.	12,000 ± 430	21.3 ± 1.1
*Citrus limon* L.	780 ± 78	24.7 ± 2.3
*Pistacia lentiscus* L. var. *chia*	2350 ± 325	12.6 ± 1.8
*Crithmum maritimum* L.	14,800 ± 195	7.5 ± 1.3

## Data Availability

The data presented in this study are available on request from the corresponding author.
